# A Bibliometric Analysis of the Top 100 Most Cited Chronotype Research Papers

**DOI:** 10.5334/jcr.146

**Published:** 2017-01-27

**Authors:** Ray Norbury

**Affiliations:** 1 University of Roehampton, Psychology Department, Whitelands College, London, SW15 4JD, GB

**Keywords:** Chronotype, bibliometric, citation

## Abstract

Bibliometric indices are a widely used measure of research impact. The aim of the current study was to identify and characterise the top one hundred most-cited research articles in the topic of chronotype research. A search of the Thomson Reuters Web of Science database returned 974 eligible articles (published between 1990 and 2016). Citations for the 100 most-cited articles ranged between 438 and 29. The most represented journal was Chronobiology International (*n* = 30). Nearly 50% of articles originated in Germany and the U.S. The bibliometrics reported identify key publications and provide insight into trends within the topic of chronotype research.

## Introduction

Circadian rhythms drive our preferences for wakefulness, periods of activity and sleep. The distribution of the timing of circadian rhythms, or chronotypes, ranges from those individuals that prefer to wake and retire to bed early to those who prefer to go to bed late and rise late, and it is becoming increasingly clear that chronotype impacts on many aspects of life including general health, mental health, cognition and addictive behaviours [1].

Bibliometric analysis measures the number of times an article is cited in the literature and in which particular journal. A citation is received when one article uses another as a reference and the number of citations an article receives can be used as an indicator of impact (an article considered to have greater importance by the research community is more likely to be cited). In addition, citation count also forms the basis for computation of the journal impact factor (IF), which is a widely used measure of journal quality. A review of the most-cited articles within a research field can, therefore, reveal topics of current interest, novel techniques and research trends. The aim of the current study was to identify and analyse the one hundred most-cited publications in the topic of chronotype research.

## Methods

### Article identification

The most-cited chronotype research articles were identified using the Institute for Scientific Information (ISI) Web of science search tool (Thomson Reuters, New York, NY). The ISI web of science is a multidisciplinary database providing a range of bibliometric information indexing over 8500 journals from a wide range of disciplines. Using the topic search term “chronotype” and title search term “*chronotype*” combined with the Boolean operator “or” (i.e. returns the value true if either or both of the operands is true) a total of 976 journal articles published between 1990 and 2016 were identified. Searching by the topic term alone returned 842 items, whereas searching by the second term alone returned 321 items. The 100 most cited chronotype papers were identified from this list. The author reviewed the title and abstract for each article to ensure its relevance (one article was excluded in this process and replaced by the next highest article in terms of citation count).

### Article analysis

The following information for each of the top 100 most-cited articles was extracted: 1) authorship, 2) article title, 3) keywords, 4) journal title, 5) year of publication, 6) total number of citations, 7) annual citation count (total number of citations/time since publication), 8) impact factor (IF) , 9) source normalised impact per paper (SNIP), 10) impact per publication (IPP) , 11) the SCImago journal rank (SJR), 12) country of origin (based on the affiliation of the first author at time of publication) and 13) article type (original research, meta-analysis or review).

## Results

The top one hundred journal articles in the field of chronotype research are presented in Table 1. Total citation count ranged from 29 to 438 (median: 47). The top ranking article was “Life between clocks: Daily temporal patterns of human chronotypes” by T. Roenneberg, A. Wirz-Justice and M. Merrow, published in the *Journal of Biological Rhythms* in 2003. Ranking articles by annual citation count, as compared to total citation count, resulted in a change in position range of –58 to +66 (where a negative number reflects a higher ranking based on annual citation count). The article with the most annual citations (rank change –4) was “Social jetlag and obesity” by T. Roenneberg, published in *Current Biology* in 2012.

**Table 1:** The one hundred most cited chronotype articles ranked in order of total citations received. Where there is equal ranking articles in the same rank received an equal number of citations.

Ranking	Article details	Total Citations

1	Roenneberg, T. Life between clocks: Daily temporal patterns of human chronotypes. JOURNAL OF BIOLOGICAL RHYTHMS 2003; 18(1): 80–90. Keywords: Circadian; clock; human; light; sleep; chronotype	438
2	Wittmann, M. Social jetlag: Misalignment of biological and social time. CHRONOBIOLOGY INTERNATIONAL 2006; 23(1–2): 497–509. Keywords: Chronotype; morningness-eveningness; psychological wellbeing; sleep quality; smoking habits	345
3	Roenneberg, T. Epidemiology of the human circadian clock. SLEEP MEDICINE REVIEWS 2007; 11(6): 429–438. Keywords: Chronotype; sleep duration; sleep deprivation; entrainment; zeitgeber; light	293
4	Giannotti, F. Circadian preference, sleep and daytime behaviour in adolescence. JOURNAL OF SLEEP RESEARCH 2002; 11(3): 191–199. Keywords: Chronotype; sleep; daytime behaviour; adoclescence; epidemiology	276
5	Roenneberg, T. Social Jetlag and Obesity. CURRENT BIOLOGY 2012; 22(10): 939–943. Keywords: Not provided. A video abstract can be viewed here	160
6	Schmidt, C. A time to think: Circadian rhythms in human cognition. COGNITIVE NEUROPSYCHOLOGY 2007; 24(7): 755–789. Keywords: Time of day; circadian; chronotype; cognitive performance; ageing	159
7	Adan, A. Circadian Typology: A Comprehensive Review. CHRONOBIOLOGY INTERNATIONAL 2012; 29(9): 1153–1175. Keywords: Age; circadian typology; chronotype; cognitive performance; morningness-eveningness; personality; psychiatric disorder; sex; shifwork	152
8	Bailey, S. Circadian rhythmicity of cortisol and body temperature: Morningness-eveningness effects. CHRONOBIOLOGY INTERNATIONAL 2001; 18(2): 249–261. Keywords: Adult; body temperature; chronotype; circadian rhythm; human; hydrocortisone; larks; owls	146
8	Taillard, J. Morningness/eveningness and the need for sleep. JOURNAL OF SLEEP RESEARCH 1999; 8(4): 291–295. Keywords: Chronic sleep deprivation; daytime somnolence; morningness/eveningness; sleelp hygiene; sleep need; sleep/wake habits	146
10	Carrier, J. Sleep and morningness-eveningness in the ‘middle’ years of life (20–59 y). JOURNAL OF SLEEP RESEARCH 1997; 6(4): 230–237. Keywords: Age; chronotype; circadian rhythms; middle-age; morningness- eveningness; sleep	143
11	Russo, P. Sleep habits and circadian preference in Italian children and adolescents. JOURNAL OF SLEEP RESEARCH 2007; 16(2): 163–169. Keywords: Chronotype morningness; circadian preference; preadolescence; sleep-habits	114
11	Paine, S. The epidemiology of morningness/eveningness: Influence of age, gender, ethnicity, and socioeconomic factors in adults (30–49 years). JOURNAL OF BIOLOGICAL RHYTHMS 2006; 21(1): 68–76. Keywords: Morningness/eveningness; chronotype; epidemiology; age; ethnicity; gender; socioeconomic deprivation; work schedules	114
13	Brown, S. Molecular insights into human daily behavior. PROCEEDINGS OF THE NATIONAL ACADEMY OF SCIENCES OF THE UNITED STATES OF AMERICA 2008; 105(5): 1602–1607. Keywords: Chronotype; circadian; fibroblast; genetics	110
14	Zavada; A. Comparison of the Munich Chronotype Questionnaire with the Horne-Ostberg’s Morningness-Eveningness Score. CHRONOBIOLOGY INTERNATIONAL 2005; 22(2): 267–278. Keywords: Human chronotypes; Sleep; Survey; Horne-Ostberg’s Morningness-Eveningness Questionnaire; Munich Chronotype Questionnare	107
15	Adan, A. Chronotype and personality-factors in the daily consumption of alcohol and psychostimulants. ADDICTION 1994; 89(4): 455–462. Keywords: N/A	100
16	Hagenauer, M. Adolescent Changes in the Homeostatic and Circadian Regulation of Sleep. DEVELOPMENTAL NEUROSCIENCE 2009; 31(4): 276–284. Keywords: Puberty; chronotype; entrainment; seep deprivation	93
17	Goldstein, D. Time of day, intellectual performance, and behavioral problems in Morning versus Evening type adolescents: Is there a synchrony effect?. PERSONALITY AND INDIVIDUAL DIFFERENCES 2007; 42(3): 431–440. Keywords: Chronotype; Intellectual performance; Adolescence; Time of day; Synchrony effect	90
17	Roenneberg, T. The human circadian clock entrains to sun time. CURRENT BIOLOGY 2007; 17(2): R44-R45. Keywords: N/A	90
19	Mansour, H. Circadian phase variation in bipolar I disorder. CHRONOBIOLOGY INTERNATIONAL 2005; 22(3): 571–584. Keywords: Circadian; rhythm; chornotype; morningness/eveningness; bipolar disorder; schizophrenia	86
20	Taillard, J. The circadian and homeostatic modulation of sleep pressure during wakefulness differs between morning and evening chronotypes. JOURNAL OF SLEEP RESEARCH 2003; 12(4): 275–282. Keywords: alertness; circadian rhythms; electroencephalogram; homeostatic process; morningness/eveningness; spectral analysis	84
21	Levandovski, R. Depression Scores Associate With Chronotype and Social Jetlag in a Rural Population. CHRONOBIOLOGY INTERNATIONAL 2011; 28(9): 771–778. Keywords: Chronotype; circadian clock; depression; mood disorders; sleep-wake behaviour; social jetlag	83
22	Lehnkering, H. Influence of chronotype, season, and sex of subject on sleep behavior of young adults. CHRONOBIOLOGY INTERNATIONAL 2007; 24(5): 875–888. Keywords: Chronotype; sex; season; sleep behaviour; wrist actigraphy	80
23	Gaspar-Barba, E. Depressive symptomatology is influenced by chronotypes. JOURNAL OF AFFECTIVE DISORDERS 2009; 119(1–3): 100–106. Keywords: Depression; Circadian rhythms; Eveningness–morningness	78
24	Garaulet, M. Timing of food intake predicts weight loss effectiveness. INTERNATIONAL JOURNAL OF OBESITY 2013; 37(4): 604–611. Keywords: Timing of food intake; weight loss; dietary treatment; circadian	77
25	Randler, C. Morningness-Eveningness Comparison in Adolescents from Different Countries around the World. CHRONOBIOLOGY INTERNATIONAL 2008; 25(6): 1017–1028. Keywords: Chronotype; circadian rhythm; climate; latitude; longitude	73
26	Mongrain, V. Circadian and homeostatic sleep regulation in morningness-eveningness. JOURNAL OF SLEEP RESEARCH 2006; 15(2): 162–166. Keywords: N/A	72
27	Hidalgo, M. Relationship between depressive mood and chronotype in healthy subjects. PSYCHIATRY AND CLINICAL NEUROSCIENCES 2009; 63(3): 283–290. Keywords: Chronobiology; chronotype; circadian rhythm; depressive mood; morningness.	71
28	Urban, R. Morningness-eveningness, chronotypes and health-impairing behaviors in adolescents.. CHRONOBIOLOGY INTERNATIONAL 2011; 28(3): 238–47. Keywords: Adolescent; Alcohol; Chronotype; Morningness-eveningness; Physical inactivity; Smoking	69
29	Monk, T. Morningness-eveningness and lifestyle regularity. CHRONOBIOLOGY INTERNATIONAL 2004; 21(3): 435–443. Keywords: N/A	67
30	Kitamura, S. Evening preference is related to the incidence of depressive states independent of sleep-wake conditions. CHRONOBIOLOGY INTERNATIONAL 2010; 27(9–10): 1797–1812. Keywords: Chronotype; circadian rhythms; depression; eveningess; neuropsychopharmacology; sleep	66
31	Caci, H. Comparing three morningness scales: Age and gender effects, structure and cut-off criteria. SLEEP MEDICINE 2009; 10(2): 240–245. Keywords: Circadian rhythms; sleep; chronotype; morningness; age; gender; factor analysis; exploratory	63
31	Kudielka, B. Morningness and eveningness: The free cortisol rise after awakening in early birds and night owls. BIOLOGICAL PSYCHOLOGY 2006; 72(2): 141–146. Keywords: Morningness–eveningness; Salivary cortisol; Cortisol awakening rise (CAR); Horne and Östberg Owl-and-Lark-Questionnaire; Hypothalamus–pituitary–adrenal axis (HPA axis) activity; Circadian rhythm	63
33	Medeiros, A. The relationships between sleep-wake cycle and academic performance in medical students. BIOLOGICAL RHYTHM RESEARCH 2001; 32(2): 263–270. Keywords: Sleep; Sleep duration; Medical students; Academic performance School	60
34	Hansen, J. Nested case-control study of night shift work and breast cancer risk among women in the Danish military. OCCUPATIONAL AND ENVIRONMENTAL MEDICINE 2012; 69(8): 551–556. Keywords: N/A	58
34	Gamble, K. Shift Work in Nurses: Contribution of Phenotypes and Genotypes to Adaptation. PLOS ONE 2011; 6(4). Keywords: N/A	58
36	Wright, K. Entrainment of the Human Circadian Clock to the Natural Light-Dark Cycle. CURRENT BIOLOGY 2013; 23(16): 1554–1558. Keywords: N/A	57
37	Roenneberg, T. Entrainment of the human circadian clock. COLD SPRING HARBOR SYMPOSIA ON QUANTITATIVE BIOLOGY 2007; 72(): 293–299. Keywords: N/A	56
38	Pedrazzoli, M. Clock polymorphisms and circadian rhythms phenotypes in a sample of the Brazilian population. CHRONOBIOLOGY INTERNATIONAL 2007; 24(1): 1–8. Keywords: Clock;Horne-Ostberg morningness–eveningness score; Circadian chronotype; Genetics of circadian rhythms; Delayed sleep phase syndrome	55
39	Selvi, Y. Mood changes after sleep deprivation in morningness-eveningness chronotypes in healthy individuals. JOURNAL OF SLEEP RESEARCH 2007; 16(3): 241–244. Keywords: Circadian; mood; shift work; sleep deprivation	54
39	Killgore, W. Effects of sleep deprivation and morningness-eveningness traits on risk-taking. PSYCHOLOGICAL REPORTS 2007; 100(2): 613–626. Keywords: N/A	54
39	Reilly, T. Diurnal variation in temperature, mental and physical performance, and tasks specifically related to football (soccer). CHRONOBIOLOGY INTERNATIONAL 2007; 24(3): 507–519. Keywords: Body temperature; Circadian rhythms; Sport performance; Soccer	54
42	Randler, C. Morningness-eveningness, sleep-wake variables and big five personality factors. PERSONALITY AND INDIVIDUAL DIFFERENCES 2008; 45(2): 191–196. Keywords: Big-five-inventory; Chronotype; Gender differences; Morningness–eveningness; Personality	53
43	Randler, C. Morningness-eveningness, habitual sleep-wake variables and cortisol level. BIOLOGICAL PSYCHOLOGY 2010; 85(1): 14–18. Keywords: Adolescents; Awakening cortisol response; Chronotype; Circadian rhythm; Morningness–eveningness	52
43	Atkinson, G. Diurnal variation in cycling performance: Influence of warm-up. JOURNAL OF SPORTS SCIENCES 2005; 23(3): 321–329. Keywords: Clinical trial; publication bias	52
45	Selvi, Y. Associations between chronotype, sleep quality, suicidality, and depressive symptoms in patients with major depression and healthy controls. CHRONOBIOLOGY INTERNATIONAL 2010; 27(9–10): 1813–1828. Keywords: Chronotype; Depression; Morningness-eveningness; Sleep quality; Suicide	51
46	Hofstra, W. How to assess circadian rhythm in humans: A review of literature. EPILEPSY & BEHAVIOR 2008; 13(3): 438–444. Keywords: Circadian rhythm; Epilepsy; Desynchrony protocol; Constant routine protocol; Dim light melatonin onset; Core body temperature; Cortisol; Sleep parameters; Actigraphy; Chronotype	50
46	Randler, C. Differences in sleep and circadian preference between Eastern and Western German adolescents. CHRONOBIOLOGY INTERNATIONAL 2008; 25(4): 565–575. Keywords: Chronotype; Circadian rhythm; Longitude; Morningness-eveningness; Sunlight	50
46	Monk, T. Measuring sleep habits without using a diary: The sleep timing questionnaire. SLEEP 2003; 26(2): 208–212. Keywords: Circadian rhythms; sleep; human; diary; ratings	50
49	Wirz-Justice, A. Diurnal variation of depressive symptoms.. DIALOGUES IN CLINICAL NEUROSCIENCE 2008; 10(3): 337–43. Keywords: Major depressive disorder; mood; circadian rhythm; sleep regulation; sleep deprivation; synchronization	48
50	Digdon, N. College Students Who Have an Eveningness Preference Report Lower Self-Control and Greater Procrastination. CHRONOBIOLOGY INTERNATIONAL 2008; 25(6): 1029–1046. Keywords: Circadian preference; College students; Self-regulation; Morningness-eveningness; Self-control	47
50	Refinetti, R. Variability of diurnality in laboratory rodents. JOURNAL OF COMPARATIVE PHYSIOLOGY A-NEUROETHOLOGY SENSORY NEURAL AND BEHAVIORAL PHYSIOLOGY 2006; 192(7): 701–714. Keywords: Circadian rhythm; diurnality; locomotor activity; arvicanthis niloticus; meriones unguiculatus; mesocricetus auratus; mus musculus; octodon degus; phodopus sungorus; rattus norvegicus	47
50	Takeuchi, H. Parental enforcement of bedtime during childhood modulates preference of Japanese junior high school students for eveningness chronotype. CHRONOBIOLOGY INTERNATIONAL 2001; 18(5): 823–829. Keywords: Adolescence; Discipline on bedtime; Gender differences; Japanese children; Junior high school student; Morningness-eveningness	47
53	Besoluk, S. Morningness-Eveningness Preferences and Academic Achievement of University Students. CHRONOBIOLOGY INTERNATIONAL 2011; 28(2): 118–125. Keywords: Achievement; Circadian rhythm; GPA; Morningness-eveningness preference; Teaching time of the day	46
53	Randler, C. Associations among Sleep, Chronotype, Parental Monitoring, and Pubertal Development among German Adolescents. JOURNAL OF PSYCHOLOGY 2009; 143(5): 509–520. Keywords: chronotype; circadian rhythm; diurnal preference; morningness-eveningness; puberty	46
53	Ong, J. Characteristics of insomniacs with self-reported morning and evening chronotypes.. JOURNAL OF SLEEP MEDICINE 2007; 3(3): 289–94. Keywords: Insomnia; chronotype; morningness; eveningness	46
56	Di Milia, L. Reviewing the Psychometric Properties of Contemporary Circadian Typology Measures. CHRONOBIOLOGY INTERNATIONAL 2013; 30(10): 1261–1271. Keywords: Chronotype; circadian typology; morningness-eveningness preference; psychometric	45
56	Olds, T. Sleep Duration or Bedtime? Exploring the Relationship between Sleep Habits and Weight Status and Activity Patterns. SLEEP 2011; 34(10): 1299–1307. Keywords: Child; screen time; physical activity; bedtime; wake up time	45
56	Randler, C. Association between morningness-eveningness and mental and physical health in adolescents. PSYCHOLOGY HEALTH & MEDICINE 2011; 16(1): 29–38. Keywords: Adolescents; chronotype; circadian preference; depression; mental health; morningness–eveningness; physical health; quality of life	45
56	Fleig, D. Association between chronotype and diet in adolescents based on food logs. EATING BEHAVIORS 2009; 10(2): 115–118. Keywords: Circadian typology; Diet; Diurnal preference; Eating behaviour; Food logs; Nutrition	45
56	Randler, C. Psychometric properties of the German version of the Composite Scale of Morningness. BIOLOGICAL RHYTHM RESEARCH 2008; 39(2): 151–161. Keywords: Age; circadian rhythms; chronotype; gender; German Composite Scale of Morningness; morningness – eveningness	45
61	Merikanto, I. Associations of Chronotype and Sleep With Cardiovascular Diseases and Type 2 Diabetes. CHRONOBIOLOGY INTERNATIONAL 2013; 30(4): 470–477. Chronotype; Circadian; Diurnal; Eveningness; Glucose tolerance; Morningness; Waist circumference	44
61	Ahn, Y. Chronotype distribution in bipolar I disorder and schizophrenia in a Korean sample. BIPOLAR DISORDERS 2008; 10(2): 271–275. Keywords: Bipolar disorder; chronotype; schizophrenia	44
63	Tzischinsky, O. Eveningness, Sleep Patterns, Daytime Functioning, and Quality of Life in Israeli Adolescents. CHRONOBIOLOGY INTERNATIONAL 2011; 28(4): 338–343. Keywords: Adolescence, Chronotype; Depressed mood; Morningness-eveningness; Quality of life; Sleep patterns; Sleep-problem behaviours; Sleepiness	43
64	Tonetti, L. Morningness-eveningness preference and sensation seeking. EUROPEAN PSYCHIATRY 2010; 25(2): 111–115. Keywords: Sensation seeking; Circadian typology; Gender; University students; Chronotype; Chronobiology	42
65	Randler, C. Validation of the full and reduced Composite Scale of Morningness. BIOLOGICAL RHYTHM RESEARCH 2009; 40(5): 413–423. Keywords: Chronotype; circadian typology; questionnaire; short form; validation	41
65	Waterhouse, J. Identifying some determinants of jet lag, and its symptoms: a study of athletes and other travellers. BRITISH JOURNAL OF SPORTS MEDICINE 2002; 36(1): 54–60. Keywords: N/A	40
67	Merikanto, I. Relation of Chronotype to Sleep Complaints in the General Finnish Population. CHRONOBIOLOGY INTERNATIONAL 2012; 29(3): 311–317. Keywords: Circadian; Eveningness; Hypnotics; Insomnia; Morningness	40
67	Allebrandt, K. CLOCK Gene Variants Associate with Sleep Duration in Two Independent Populations. BIOLOGICAL PSYCHIATRY 2010; 67(11): 1040–1047. Keywords: CLOCK; sleep duration; clock genes; MCTQ; short sleepers; long sleepers	40
67	Jankowski, K. Diurnal variation in energetic arousal, tense arousal, and hedonic tone in extreme morning and evening types. CHRONOBIOLOGY INTERNATIONAL 2008; 25(4): 577–595. Keywords: Chronotype; MEQ; Diurnal rhythm; Mood	40
70	Preckel, F. Chronotype, cognitive abilities, and academic achievement: A meta-analytic investigation. LEARNING AND INDIVIDUAL DIFFERENCES 2011; 21(5): 483–492. Keywords: Chronotype; Morningness–eveningness; Meta-analysis; Cognitive ability; Academic achievement; Grades; Prediction	39
71	Borisenkov, M. Chronotype, sleep length, and school achievement of 11-to 23-year-old students in northern european russia. CHRONOBIOLOGY INTERNATIONAL 2010; 27(6): 1259–1270. Keywords: Chronotype; Circadian rhythm; College students; Northern latitudes; School achievements; School children; Sleep length	38
71	Werner, H. Assessment of Chronotype in Four- to Eleven-Year-Old Children: Reliability and Validity of the Children’s ChronoType Questionnaire (CCTQ). CHRONOBIOLOGY INTERNATIONAL 2009; 26(5): 992–1014. Keywords: Chronotype; Midsleep point; Morningness/eveningness; Validity; Children	38
71	Cuninkova, L. Peripheral circadian oscillators - Interesting mechanisms and powerful tools. MOLECULAR AND BIOPHYSICAL MECHANISMS OF AROUSAL, ALERTNESS, AND ATTENTION 2008; 1129(): 358–370. Keywords: Circadian; clock; peripheral; chronotype; genetics	38
74	Vollmer, C. Outdoor Light at Night (LAN) Is Correlated With Eveningness in Adolescents. CHRONOBIOLOGY INTERNATIONAL 2012; 29(4): 502–508. Keywords: Adolescents; Chronotype; CSM; Electronic screen media; Intake of stimulants; Light at night (LAN); Light pollution; Midpoint of sleep; Remote sensing	37
74	Mongrain, V. Chronotype and sex effects on sleep architecture and quantitative sleep EEG in healthy young adults. SLEEP 2005; 28(7): 819–827. Keywords: Morningness-eveningness; sleep; spectral analysis; circadian rhythms; human; sex difference; sleep regulation	37
74	Challet, E. Circadian organization in a diurnal rodent, Arvicanthis ansorgei Thomas 1910: Chronotypes, responses to constant lighting conditions, and photoperiodic changes. JOURNAL OF BIOLOGICAL RHYTHMS 2002; 17(1): 52–64. Keywords: Locomotor activity; rhythm; period; phase; day length; entrainment; Arvicanthis	37
74	Labyak, S. Rhythm chronotypes in a diurnal rodent, Octodon degus. AMERICAN JOURNAL OF PHYSIOLOGY-REGULATORY INTEGRATIVE AND COMPARATIVE PHYSIOLOGY 1997; 273(3): R1058-R1066. Keywords: N/A	37
78	Jonason, P. Creatures of the night: Chronotypes and the Dark Triad traits. PERSONALITY AND INDIVIDUAL DIFFERENCES 2013; 55(5): 538–541. Keywords: Dark Triad; Narcissism; Psychopathy; Machiavellianism; Morningness: eveningness; Chronotype; Evolutionary psychology	34
78	Reutrakul, S. Chronotype Is Independently Associated With Glycemic Control in Type 2 Diabetes. DIABETES CARE 2013; 36(9): 2523–2529. Keywords: N/A	34
78	Allebrandt, K. A K-ATP channel gene effect on sleep duration: from genome-wide association studies to function in Drosophila. MOLECULAR PSYCHIATRY 2013; 18(1): 122–132.Keywords: N/A	34
78	Hsu, C. Associations Between Chronotypes, Psychopathology, and Personality Among Incoming College Students. CHRONOBIOLOGY INTERNATIONAL 2012; 29(4): 491–501. Keywords: Chronotypes; Evening type; Morningness-eveningness; Personality; Psychopathology; Young adults	34
78	Di Milia, L. Demographic factors; fatigue, and driving accidents: An examination of the published literature. ACCIDENT ANALYSIS AND PREVENTION 2011; 43(2): 516–532. Keywords: Fatigue; Demographics; Driving accidents; Working arrangement; Circadian chronotype; Personality traits	34
78	Wittmann, M. Decreased Psychological Well-Being in Late ‘Chronotypes’ Is Mediated by Smoking and Alcohol Consumption. SUBSTANCE USE & MISUSE 2010; 45(1–2): 15–30. Keywords: Cigarette; smoking; alcohol; chronotype; sleep-wake cycle; mood	34
78	Caci, H. Inattentive Symptoms of ADHD Are Related to Evening Orientation. JOURNAL OF ATTENTION DISORDERS 2009; 13(1): 36–41. Keywords: Attention; impulsivity; hyperactivity; attention-deficit/hyperactivity disorder; circadian rhythms; chronotype; morningness; factor analysis; exploratory	34
85	Osland, T. Association Study of a Variable-Number Tandem Repeat Polymorphism in the Clock Gene PERIOD3 and Chronotype in Norwegian University Students. CHRONOBIOLOGY INTERNATIONAL 2011; 28(9): 764–770. Keywords: Association study; Chronotype; Circadian rhythm; Diurnal preference; Genotype; Morningness/eveningness; PER3	33
85	Leonhard, C. In Sync with the Family: Children and Partners Influence the Sleep-Wake Circadian Rhythm and Social Habits of Women. CHRONOBIOLOGY INTERNATIONAL 2009; 26(3): 510–525. Keywords: Children; Circadian rhythm; Family synchronization; Parents; Partner	33
85	Rea, M. A new approach to understanding the impact of circadian disruption on human health. JOURNAL OF CIRCADIAN RHYTHMS 2008; (6) Keywords: N/A	33
88	Selvi, Y. Chronotype Differences in Suicidal Behaviour and Impulsivity Among Suicide Attempters. CHRONOBIOLOGY INTERNATIONAL 2011; 28(2): 170–175. Keywords: Biological rhythms; Chronotype; Circadian; Impulsivity; Morningness/eveningness; Suicide	32
88	Kantermann, T. Shift-work research: Where do we stand, where should we go?. SLEEP AND BIOLOGICAL RHYTHMS 2010; 8(2): 95–105. Keywords: N/A	32
88	Vernet, C. Idiopathic Hypersomnia with and without Long Sleep Time: A Controlled Series of75 Patients. SLEEP 2009; 32(6): 753–759. Keywords: Hypersomnia; long sleep time; MSLT	32
88	Mongrain, V. Increased homeostatic response to behavioral sleep fragmentation in morning types compared to evening types. SLEEP 2007; 30(6): 773–780. Keywords: Morningness-eveningness; sleep fragmentation; sleep; EEG spectral analysis; circadian rhythms; human; sleep regulation	32
88	Goulet, G. Daily light exposure in morning-type and evening-type individuals. JOURNAL OF BIOLOGICAL RHYTHMS 2007; 22(2): 151–158. Keywords: Morningness-eveningness; chronotype; light exposure; circadian phase; circadian period; sleep schedule; ambulatory recordings; entrainment	32
93	Pagani, L. The Physiological Period Length of the Human Circadian Clock In Vivo Is Directly Proportional to Period in Human Fibroblasts. PLOS ONE 2010; 5(10). Keywords: N/A	31
93	Monk, T. A sleep diary and questionnaire study of naturally short sleepers. JOURNAL OF SLEEP RESEARCH 2001; 10(3): 173–179. Keywords: N/A	31
95	Lucassen, E. Evening Chronotype Is Associated with Changes in Eating Behavior, More Sleep Apnea, and Increased Stress Hormones in Short Sleeping Obese Individuals. PLOS ONE 2013; 8(3). Keywords: N/A	30
95	Pagani, L. Serum factors in older individuals change cellular clock properties. PROCEEDINGS OF THE NATIONAL ACADEMY OF SCIENCES OF THE UNITED STATES OF AMERICA 2011; 108(17): 7218–7223. Keywords: Chronobiology; peripheral oscillators; human behaviour	30
95	Randler, C. Age and Gender Differences in Morningness-Eveningness During Adolescence. JOURNAL OF GENETIC PSYCHOLOGY 2011; 172(3): 302–308. Keywords: Morningness–eveningness; chronotype; adolescents; age; gender; Turkey	30
95	Matchock, R. Chronotype and time-of-day influences on the alerting, orienting, and executive components of attention. EXPERIMENTAL BRAIN RESEARCH 2009; 192(2): 189–198. Keywords: Alertness; attention; chronotype; conflict; diurnal; eveningness; morningness; time-of-day	30
95	Schubert, E. Association between chronotype and the constructs of the Three-Factor-Eating-Questionnaire. APPETITE 2008; 51(3): 501–505. Keywords: Circadian typology; Composite Scale of Morningness; Diet, Dietary restraint; Disinhibition; Diurnal preference; Eating behaviour; Morningness–eveningness; Perceived hunger; Three-Factor-Eating-Questionnaire	30
100	Juda, M. Chronotype Modulates Sleep Duration, Sleep Quality, and Social Jet Lag in Shift-Workers. JOURNAL OF BIOLOGICAL RHYTHMS 2013; 28(2): 141–151. Keywords: Chronotype; MCTQ shift; shift-work; circadian misalignment; social jet lag; sleep duration; sleep quality age	29

N/A = not applicable (keywords either not available or not included in the journal format).

The journal with the most publications in the top one-hundred was *Chronobiology International* (*n* = 30), followed by the *Journal of Sleep Research* (*n* = 8) and *Sleep* and *Journal of Biological Rhythms* (both, *n* = 5). The median/range IF, SNIP, IPP and SJR was, respectively, 3.11/0.53–13.31, 1.24/0.43–3.10, 3.27/0.64–10.58, 1.37/0.25–5.93 (see Table 2).

**Table 2:** Journal in which the one hundred most cited chronotype articles were published ranked by number of articles. Where there is equal ranking articles in the same rank published an equal number of articles.

Journal	No Articles	IF	SNIP	IPP	SJR

CHRONOBIOLOGY INTERNATIONAL	30	3.540	1.137	3.339	1.264
JOURNAL OF SLEEP RESEARCH	8	3.093	1.505	3.337	1.283
SLEEP	5	4.793	1.907	4.927	2.053
JOURNAL OF BIOLOGICAL RHYTHMS	5	2.824	0.961	2.824	1.553
CURRENT BIOLOGY	3	9.571	1.490	5.419	3.732
PLOS ONE	3	3.057	1.034	3.270	1.300
PERSONALITY AND INDIVIDUAL DIFFERENCES	3	1.861	1.183	2.156	1.134
BIOLOGICAL RHYTHM RESEARCH	3	0.695	0.468	0.802	0.245
PROCEEDINGS OF THE NATIONAL ACADEMY OF SCIENCES OF THE UNITED STATES OF AMERICA	2	9.423	2.542	9.238	5.781
BIOLOGICAL PSYCHOLOGY	2	3.234	1.153	3.278	1.516
MOLECULAR PSYCHIATRY	1	13.314	2.488	10.575	5.930
BIOLOGICAL PSYCHIATRY	1	11.212	2.165	8.905	4.775
DIABETES CARE	1	8.420	3.091	7.849	4.190
SLEEP MEDICINE REVIEWS	1	7.341	3.095	7.958	2.708
BRITISH JOURNAL OF SPORTS MEDICINE	1	5.025	2.008	4.072	1.596
ADDICTION	1	4.927	1.841	4.080	2.036
BIPOLAR DISORDERS	1	4.882	1.633	5.186	2.774
EUROPEAN PSYCHIATRY	1	3.912	1.000	2.500	1.341
OCCUPATIONAL AND ENVIRONMENTAL MEDICINE	1	3.745	1.650	3.129	1.401
JOURNAL OF AFFECTIVE DISORDERS	1	3.570	1.390	3.668	1.691
JOURNAL OF ATTENTION DISORDERS	1	3.384	1.020	2.351	1.046
SLEEP MEDICINE	1	3.339	1.224	3.305	1.174
AMERICAN JOURNAL OF PHYSIOLOGY-REGULATORY INTEGRATIVE AND COMPARATIVE PHYSIOLOGY	1	3.168	*	*	*
APPETITE	1	3.125	1.260	3.034	1.218
DEVELOPMENTAL NEUROSCIENCE	1	2.898	0.857	3.176	1.490
EPILEPSY & BEHAVIOR	1	2.332	*	*	*
JOURNAL OF SPORTS SCIENCES	1	2.142	0.579	2.525	1.178
EXPERIMENTAL BRAIN RESEARCH	1	2.057	1.894	2.432	1.193
JOURNAL OF COMPARATIVE PHYSIOLOGY A-NEUROETHOLOGY SENSORY NEURAL AND BEHAVIORAL PHYSIOLOGY	1	1.988	*	*	*
EATING BEHAVIORS	1	1.962	0.741	1.744	0.592
LEARNING AND INDIVIDUAL DIFFERENCES	1	1.630	1.326	1.956	1.083
COGNITIVE NEUROPSYCHOLOGY	1	1.444	0.610	1.867	1.081
PSYCHOLOGY HEALTH & MEDICINE	1	1.347	*	*	*
JOURNAL OF PSYCHOLOGY	1	1.250	*	*	*
SUBSTANCE USE & MISUSE	1	1.133	*	*	*
SLEEP AND BIOLOGICAL RHYTHMS	1	0.628	0.426	0.774	0.308
JOURNAL OF GENETIC PSYCHOLOGY	1	0.542	0.477	0.843	0.323
PSYCHOLOGICAL REPORTS	1	0.530	0.434	0.635	0.287
COLD SPRING HARBOR SYMPOSIA ON QUANTITATIVE BIOLOGY	1	*	0.915	4.183	2.934
DIALOGUES IN CLINICAL NEUROSCIENCE	1	*	1.400	4.650	1.974
JOURNAL OF CIRCADIAN RHYTHMS	1	*	0.640	1.467	0.567
JOURNAL OF SLEEP MEDICINE	1	*	*	*	*
MOLECULAR AND BIOPHYSICAL MECHANISMS OF AROUSAL, ALERTNESS, AND ATTENTION	1	*	*	*	*

For abbreviations please see main text.

**Figure 1:**
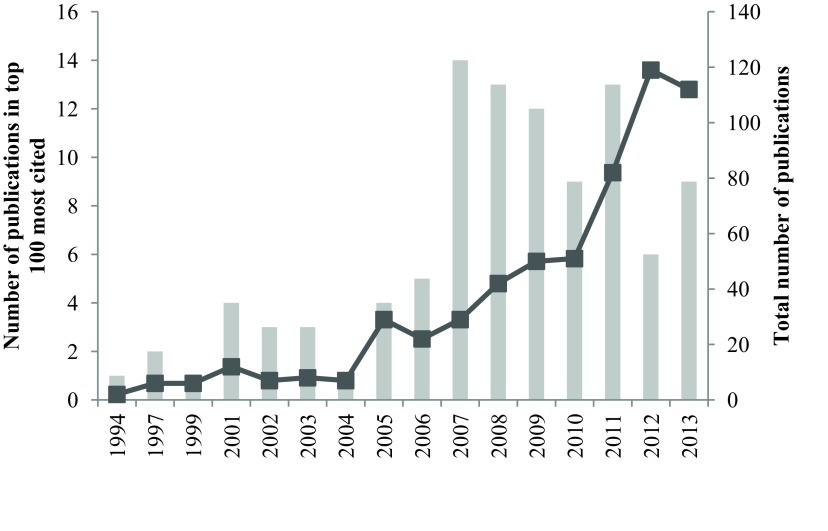
Distribution of top 100 publications by year (grey bars) and total publications per year (dark grey squares).

**Figure 2:**
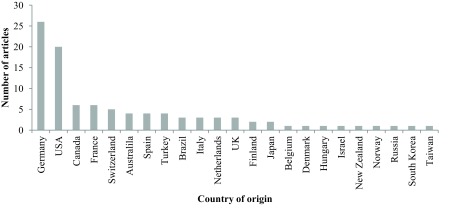
Number of publications as a function of country of origin.

Figure 1 shows the distribution of publications by year. The year with the most publications from the top one-hundred most-cited articles was 2007 (*n* = 14) (Figure 1). The most frequent first author was Randler, C (*n* = 9) (Table 3), and the majority of articles originated in Germany (*n* = 26) (Figure 2).

**Table 3:** First authors who contributed two or more of the top one hundred chronotype articles.

First Author	No Articles

Randler, C	9
Roenneberg, T	5
Mongrain, V	3
Monk, T	3
Selvi, Y	3
Adan, A	2
Allebrandt, K	2
Caci, H	2
Di Milia, L	2
Merikanto, I	2
Pagani, L	2
Taillard, J	2
Wittmann, M	2

The most frequent article type was original research (*n* = 89) and of these 54 (61%) were entirely questionnaire based. Ten review articles and one meta-analysis were also included in the top 100 most-cited articles.

## Discussion

Bibliometrics are frequently used in the library and information sciences to provide quantitative analysis of academic literature. This approach can be used to explore the impact of a field or research discipline, the impact of an individual/group of researchers or the impact of a particular paper.

The top one-hundred most cited articles in the field of chronotype were cited between 438 and 29 times. Annual citation count was also considered to evaluate current impact of an article. Although there were a number of substantial shifts in ranking, the articles with the top 5 *annual* citation counts were also in the top seven for *total* citation count thereby suggesting both historical importance and current relevance.

Perhaps not surprisingly, nearly 50% of the most-cited papers were published in discipline-related journals (*Chronobiology International, Journal of Sleep Research, Sleep and Journal of Biological Rhythms, Biological Rhythm Research*). The remaining publications encompassed a broad range of disciplines (e.g. psychology, psychiatry, nutrition and addiction) suggesting a wide audience and broad appeal of chronotype research.

With regard to year of publication, an interesting pattern of results was revealed. Seventy-six percent of the most cited articles were published post 2006 (Figure 1). The large increase in most-cited publications from 2007 onwards was mirrored (with a lag) by an equally sharp increase in total publications per year. The broad range of journal disciplines represented in the top one hundred most cited articles and the surge in publication rate suggest expanding interest in chronotype generally and increasing awareness of the impact of chronotype on a broad range of factors including cognitive function, general health and psychopathology.

Over a quarter of the top one hundred most cited articles originated from Germany. It seems unlikely that this can be attributed to the size of the scientific community or underpinning resources as this number outstripped the U.S. Rather, this figure appears to reflect the work of two principal investigators, Professor Till Roenneberg (Institute of Medical Psychology, Ludwig-Maximilian University, Munich) and Christoph Randler (University of Tübingen).

Lastly, the majority of most-cited articles represented original research, of which 61% were entirely questionnaire based. There appears to be scope to draw from other research disciplines and include a broader range of techniques and methodologies (e.g. neuroimaging, endocrinology and genetics).

Blibliometric analyses can provide insight into the development of a research field and highlight patterns, strengths and research trends over time. The approach is not without its limitations, however, and these should be taken into consideration when interpreting the current data. For example, total citation count may preferentially bias older articles. To account for this, a time adjusted metric (annual citations) was also reported. Second, the articles identified for inclusion are dependent on the search parameters selected. In the current study only publications from 1990 onwards were included and the search term was restricted to chronotype. The choice of search parameters may, therefore, exclude influential papers (e.g. Horne, J. A., & Östberg, O. (1976). A self-assessment questionnaire to determine morningness-eveningness in human circadian rhythms. *International Journal of Chronobiology, 4*(2), 97–110). Further considerations are obliteration by incorporation (influential research subsumed into common knowledge and therefore not cited), omission-bias (ignoring contradictory results) and self-citation. Finally, a direct comparison of the top citation counts across fields or disciplines is challenging and may detract from the importance of individual contributions to a small, specialised field of research. For example, the most cited genetics article had 22,085 citations. A search using the term sleep revealed a top-cited article with 6,365 citations. Nevertheless, the data presented in this report suggest that interest in chronotype research is increasing. Future chronotype citation reports may benefit from the inclusion of altmetrics that allows a broader analysis of the use of scholarly articles and includes not only citations but views, downloads etc.

In conclusion, the importance of assessing impact (on both the research field and the broader general public) is becoming increasingly important, and many research-funding bodies use impact as a means to justify their spending to governments and donors. The bibliometic analysis reported here included the top one hundred most cited papers in the topic of chronotype research. The data provide insight into the impact of chronotype research, the relative importance of a journal within the field (IF), the impact of a particular article (as indexed by citation count) and the contributions by specific researchers or groups.
